# Electrocortical Correlates of Emotion Processing and Resilience in Individuals with Adverse Childhood Experiences

**DOI:** 10.1007/s40653-024-00621-w

**Published:** 2024-03-20

**Authors:** Stephanie D. Clarke, Diana K. Riser, Mark S. Schmidt

**Affiliations:** 1 Department of Psychology, Columbus State University, 4225 University Ave., Columbus, GA 31907 USA; 2 Mercer University School of Medicine, 1250 E 66th St, Savannah, GA 31404 USA

**Keywords:** Childhood trauma, Emotion, Resilience, Evoked potentials

## Abstract

Childhood trauma is associated with poor health outcomes in adulthood, largely due to the impact of chronic stress on the body. Fortunately, there are certain protective characteristics, such as *constraint* (i.e., impulse control, inhibition, and avoidance of unconventional behavior and risk) and *cognitive reappraisal* (i.e., reframing circumstances in a more positive light). In the present study, we investigated the interaction between childhood trauma, resilience, and neural correlates of emotion processing. Participants responded to survey questions regarding childhood trauma and resilient characteristics. They were later invited to passively view neutral, unpleasant, and pleasant images while their brain activity was recorded via electroencephalography (EEG). We analyzed two event-related potential (ERP) components of interest: the Early Posterior Negativity (EPN) and Late Positive Potential (LPP). We found that childhood trauma was associated with decreased constraint and reduced sensitivity to unpleasant images (i.e., decreased LPP amplitude differences between neutral and unpleasant images as compared to controls). Further, constraint predicted increased sensitivity to pleasant images. In a hierarchical linear regression analysis, we found that constraint moderated the relation between childhood trauma and emotion processing, such that it predicted increased sensitivity to unpleasant images for adults with childhood trauma in particular. Childhood trauma and cognitive reappraisal independently predicted decreased sensitivity to unpleasant images, (i.e., decreased LPP amplitude differences between neutral and unpleasant images). Our findings suggest that childhood trauma and resilient characteristics independently and interactively influence emotion processing.

## Introduction

Childhood trauma has been shown to predict numerous poor health outcomes in adulthood, including depression, cancer, obesity, and heart disease (Felitti et al., 1998; Hughes et al., 2017). Meta-analytic evidence shows that in addition to poor health, childhood trauma predicts maladaptive coping behaviors such as sexual risk taking, alcohol and drug abuse, suicide, and inter- and intra-personal violence (Hughes et al., 2017). Hughes and colleagues (2017) suggest that these outcomes may ultimately stem from the damaging impact that chronic early life stress has on nervous, endocrine, and immune system function. However, some individuals appear to be resilient in the face of trauma, exhibiting no appreciable health detriments (Jackson, 2014). According to Denckla et al. (2020), “resilience is best conceptualized as a multi-level, dynamic process of adaptation to stress and trauma exposure.” We sought to investigate whether certain resilient characteristics predict neural correlates of emotion processing in adults with a history of childhood trauma. In particular, we investigated whether *constraint* (i.e., impulse control, avoidance of unconventional behavior and risk) and *cognitive reappraisal* (i.e., the practice of reevaluating one’s circumstances in a more positive light) moderated the relation between childhood trauma and electrocortical responses to emotional images in a sample of college students.

### Childhood Trauma and Event-Related Potentials

We examined neural correlates of emotion processing using *event-related potentials* (ERPs). ERPs are averages of electrical potentials produced by the brain, recorded via electroencephalography (EEG), that specifically reflect its response to target stimuli or events (Luck, 2012). ERPs are often linked to emotional and cognitive processes by recording participants’ brain activity as they engage in psychological tasks. In the present study, data of interest included *amplitudes*, or the magnitude of the ERP *component.* According to Luck (2012), a component is “a voltage deflection that is produced when a specific neural process occurs in a specific brain region. Many components will be elicited by a stimulus in a given task, and the different components sum together to produce the observed ERP waveform” (p. 2).

One way to measure affective discrimination using ERPs is to find the amplitude difference between participants’ responses to low-arousing, neutral images and high-arousing, affective images (Luck, 2012). Essentially this creates a *difference wave* thought to reflect affective discrimination, with larger amplitudes typically reflecting a greater sensitivity to affective as compared to neutral stimuli. A smaller amplitude difference wave, on the other hand, indicates that sensitivity to neutral and affective stimuli are more comparable and that affective discrimination is reduced. This may be adaptive or maladaptive depending on the context (Gluckman et al., 2007). For example, reduced affective discrimination may be a marker of *anhedonia* (i.e., low mood and diminished pleasure or arousal in response to evocative stimuli) and may protect against the damaging effects of chronic stress on the body by reducing the stress response in environments replete with stressors. However, in safe environments, this protective mechanism may do more harm than good by suppressing the pleasure response and decreasing *eustress*, acute stress with positive effects (e.g., stress experienced during exercise, social interactions, or new experiences). For example, Heo et al. (2021) found that individuals with a history of childhood trauma with blunted baseline reward reactivity were at the greatest risk for major depressive disorder following the COVID-19 pandemic; they also displayed reduced response inhibition in a go/no-go task, and reduced electrocortical responses to stimuli in general. Thus, larger amplitude difference waves in response to pleasant stimuli and smaller amplitude difference waves in response to unpleasant stimuli may be indicative of adaptive coping and resilience in adults with a history of childhood trauma.

In the present study, we recorded participants’ EEG as they passively viewed neutral and affective images from the International Affective Pictures System (IAPS; Lang et al., 1997). IAPS images include high-arousing (i.e., pleasant and unpleasant) and low-arousing (i.e., neutral) images that reliably evoke difference waves in healthy individuals (Dolcos & Cabeza, 2002). Thus, we used difference waves as a measure of affective discrimination. Larger amplitude difference waves reflect increased distinction between affective and neutral stimuli, and smaller amplitude difference waves reflect reduced distinction between affective and neutral stimuli.

We were particularly interested in two ERP components thought to capture aspects of emotional processing–the *Early Posterior Negativity* (EPN) and the *Late Positive Potential* (LPP) (Luck, 2012). The EPN is a negative component found over the visual cortex that peaks around 200 ms post-stimulus (Luck, 2012) within a time window 175–275 ms post-stimulus (Weinberg et al., 2015). The EPN may reflect early visual attention and activity from the fight-or-flight regions of the brain (Simonetti, 2020). The LPP is a positive component that peaks around 300 ms post-stimulus (Luck, 2012). According to Weinberg et al. (2015), information regarding the time-course of emotion processing may be gathered from analyzing the LPP in multiple time windows, specifically between 300 and 600 ms, 600–1000 ms, and 1000–1500 ms post-stimulus. The LPP begins in centro-parietal regions and shifts to a more diffuse frontal distribution in later stages of emotion processing (Weinberg et al., 2015). The LPP may be a marker of more controlled emotion processing and working memory; it is associated with allocation of attention to emotional stimuli. It is evoked in conjunction with activity in regions that drive higher-order processes, such as the prefrontal and parietal cortices, as well as emotion centers of the brain, such as the anterior cingulate cortex, amygdala, and anterior insula (Simonetti, 2020). The EPN and LPP components were used in the present study as neural correlates of emotion processing in real-time as individuals with different levels of childhood trauma viewed affective images.

### The EPN and Childhood Trauma

Few studies have specifically explored the relation between childhood trauma and the EPN component. Those that have largely used emotional faces as stimuli (e.g., Chu et al., 2016; Cicchetti & Curtis, 2005; Curtis & Cicchetti, 2011). Toddlers with a history of childhood trauma, for example, have shown atypical processing of emotional and neutral faces (Cicchetti & Curtis, 2005; Curtis & Cicchetti, 2011). In Cicchetti and Curtis’s (2005) study, toddlers with a history of childhood trauma showed the largest difference in peak amplitudes between angry and neutral faces. In Curtis and Cicchetti’s (2011) later study, toddlers with a history of childhood trauma displayed *attenuated* (i.e., smaller) amplitudes overall (i.e., in response to neutral, happy, and angry faces) at occipital sites as compared to controls. A more recent study found a relation between a greater number of traumatic events experienced and attenuated EPN difference waves in response to pleasant versus neutral IAPS images, which include both people and scenes (Sill et al., 2020). This was found over parieto-occipital sites, starting around 150 ms post-stimulus and reaching maximum amplitude difference between 200 and 300 ms post-stimulus. In the present study, participants were presented with neutral, pleasant, and unpleasant IAPS images to replicate this effect. We expected childhood trauma to predict reduced affective discrimination (i.e., attenuated EPN difference wave amplitudes) between affective and neutral images.

### The LPP and Childhood Trauma

The LPP is implicated in more controlled emotion processing and, similar to the EPN, is typically suppressed in adults with a history of childhood trauma. However, this is a general trend, as LPP patterns in adults with a history of childhood trauma seem to depend on the type and severity of trauma experienced, as well as the type of stimuli presented.

As an example, physically abused children show heightened stress responses and increased affective discrimination between neutral and threatening stimuli. Shackman et al. (2007) found that physically abused children showed heightened LPP responses to angry faces as compared to controls, particularly after viewing pictures of their own angry mothers, at frontoparietal sites 300–500 ms and 570–770 ms post-stimulus. In a go/no-go emotional paradigm, Pollack et al. (1997) found that maltreated children’s LPP amplitudes 450–500 ms and 585–635 ms post-stimulus were significantly attenuated in response to happy target stimuli as compared to non-maltreated children, despite similar task performance. Pollack et al. (2001) later found that maltreated children responded more accurately and had larger amplitude LPP responses to angry oddball stimuli than non-maltreated children in oddball tasks using emotional faces as stimuli. In sum, it seems that maltreated children are especially sensitive to threatening stimuli, but less sensitive to pleasant stimuli.

Pincham et al. (2016) found that adolescents with a history of childhood trauma initially displayed impaired *affective discrimination*, measured by the difference in LPP amplitudes in response to emotional versus neutral images as compared to control populations. However, with more than nine months of therapeutic intervention aimed at improving emotional coping strategies, the amplitudes of the participant’s LPP responses to affective images increased to be more similar to that of controls. Specifically, in the 400–700 ms time window post-stimulus presentation, unpleasant images evoked larger responses for the extended-intervention group (i.e., more than nine months of intervention) but not the minimal-intervention group (i.e., less than four months of intervention), and in the 700–1000 ms time window post-stimulus, the same pattern emerged. Individuals with a history of childhood trauma may be less sensitive to emotional stimuli than controls, but responses to these stimuli may normalize with long-term intervention. Pincham et al. (2019) found that, in a go/no-go task where participants were told they would receive performance-based feedback, but actually received fixed feedback unrelated to task performance, at-risk adolescents who had undergone minimal therapeutic intervention showed attenuated LPP difference wave amplitudes between win versus loss trials as compared to at-risk adolescents who had undergone extended therapeutic intervention. This further supports the notion that childhood trauma is related to reduced affective discrimination, or attenuated responses to pleasant stimuli and/or enhanced response to unpleasant stimuli.

Superior performance by maltreated children in go/no-go and oddball tasks with angry faces as targets suggests that changes in emotion processing due to childhood trauma may be adaptive in threatening social environments. However, this variation seems to be sensitive to therapeutic intervention, as shown in Pincham et al.’s (2016) study. Lecei and Winkel (2020) note that childhood adversity is associated with hippocampus and amygdala abnormalities, which are associated with impaired pattern separation of emotional information and thus increased fear generalization. Affective discrimination may be a useful electrocortical index of pattern separation. We expected participants with a history of childhood trauma in our study to display a similar pattern of brain activity–reduced affective discrimination in response to affective stimuli except, perhaps, those that could be perceived as angry or threatening. However, we did not analyze data for angry images, specifically, and thus expected our participants with a history of childhood trauma to show reduced affective discrimination for both the EPN and LPP in response to pleasant and unpleasant images more generally. Our next goal was to determine whether resilient characteristics have the potential to change this trajectory of emotion processing in adults with a history of childhood trauma.

### Emotion Processing and Resilience

In sum, ERP research has shown that childhood trauma is associated with increased EPN and LPP amplitudes in response to angry or threatening stimuli, but decreased EPN and LPP amplitudes in response to affective and neutral stimuli overall as compared to controls. However, there are several questions based on past research that remain unanswered. For example, it is not entirely clear whether the ERP pattern may change from childhood to adulthood. Further, Pincham et al. (2016) found that therapeutic intervention changed the brain activity of at-risk adolescents to be more similar to that of controls. It is thus possible that adults with a history of childhood trauma exhibit divergent ERP patterns depending on their emotional coping strategies. Thus, the present study sought to determine whether psychological surveys designed to assess emotion regulation strategies could be used to predict divergent ERP patterns in adults with a history of childhood trauma.

### Cognitive Reappraisal

*Cognitive reappraisal* (i.e., evaluating unpleasant or aversive stimuli in a more positive light) is one emotion regulation strategy that may be implicated in resilience. In a 2022 systematic review, Riepenhausen et al. (2022) found a clear relation between cognitive reappraisal and increased positive affect, self-reported resilience, well-being, self esteem, life satisfaction, self-efficacy, autonomy, closeness and positive relations with others, and emotion regulation abilities. Notably, this relation was stronger among those who reported high levels of adversity or exposure to stressors, and people with childhood trauma who reported high levels of cognitive reappraisal demonstrated increased resilience through various measures. In a review by MacNamara et al. (2022), cognitive reappraisal is described as the gold standard of emotion regulation. Harrison and Chassy (2019) found that participants who scored higher in cognitive reappraisal displayed smaller LPP amplitudes 1000–1500 ms post-stimulus in response to threatening images as compared to participants who scored lower in cognitive reappraisal. Cauwenberge et al. (2017) found that children’s LPPs appeared to have smaller amplitudes when the children were shown unpleasant IAPS pictures that were coupled with neutral, reappraising explanations as opposed to negative explanations. This suggests that cognitive reappraisal may predict the emotional response to unpleasant images. Langeslag and Strien (2018) also found that when participants practiced using cognitive reappraisal while viewing images of snakes and spiders, their LPPs reduced in amplitude but not their EPNs. Participants who used cognitive reappraisal when shown pictures of their romantic ex-partners demonstrated reduced LPP amplitudes (Langeslag & Sanchez, 2018). This suggests that adaptive coping strategies such as cognitive reappraisal may be reflected as controlled, late emotion processing in the LPP, but not in the more immediate, visual attention stages that the EPN is thought to reflect. Considering this evidence, we hypothesized that high levels of reappraisal would be associated with smaller LPP responses to unpleasant images, and thus smaller difference wave amplitudes for unpleasant versus neutral stimuli.

### Constraint

*Constraint* (i.e., impulse control and avoidance of unconventional behavior and risk), is another resilient characteristic involved in emotion processing. An early study found that low constraint predicted reduced startle habituation as measured by eye-blink responses to acoustic stimuli (LaRowe et al., 2006). Sach et al. (2018) found that individuals who scored lower in constraint displayed shorter EPN latencies and reduced behavioral inhibition in a Go/No-Go task. Brennan and Baskin-Sommers (2018) found that impulsive and disinhibited real-world behavior (i.e., low constraint), as well as poor performance on an oddball task, was linked to attenuation of emotion-related ERP components. Munk et al. (2020) found that low constraint scores were associated with attenuated LPP amplitudes in response to erotic images. This may stem from a higher threshold of emotional activation amongst impulsive or low-constraint individuals, hence their sensation-seeking behaviors. Thus, constraint may be a useful predictor of both EPN and LPP amplitudes, such that low constraint predicts reduced amplitudes in controls. Thus, we predicted that individuals with high levels of constraint would show increased affective discrimination as evidenced by larger difference wave amplitudes for both the EPN and LPP than those with low levels of constraint in response to emotional images.

### Interactions between Constraint, Cognitive Reappraisal, and Childhood Trauma

In the present study, we were interested in determining whether an interaction exists between cognitive reappraisal, constraint, and electrocortical responses to emotional images. In controls, cognitive reappraisal is associated with reduced emotional responses to unpleasant stimuli and attenuated difference wave amplitudes. Constraint, on the other hand, is associated with a lower threshold of emotional activation and increased affective discrimination (i.e., enhanced difference wave amplitudes). It is then possible that constraint and cognitive reappraisal serve as protective factors in the relation between childhood trauma and maladaptive emotion processing via different mechanisms: Cognitive reappraisal may decrease negative affect, whereas constraint may increase positive affect in response to stimuli, for example. Low constraint is often associated with a higher threshold of emotional activation, which drives externalizing behaviors (impulsivity, risk-taking, sensation-seeking; Chu et al., 2016). In contrast, those with high constraint may have a lower threshold of emotional activation and little desire to engage in sensation-seeking behaviors.

The present study sought to reconcile these findings and investigate how resilient characteristics, specifically cognitive reappraisal and constraint, moderate the relation between childhood trauma and electrocortical responses to affective images. Using a sample of college adults, we hypothesized the following: (1) Cognitive reappraisal would predict decreased sensitivity to unpleasant images in the later stages of emotion processing, and therefore smaller LPP difference wave amplitudes for unpleasant images. (2) Constraint would predict increased sensitivity to affective images, and therefore larger EPN and LPP difference wave amplitudes for pleasant and unpleasant images. (3) Cognitive reappraisal would moderate the relation between childhood trauma and brain activity by decreasing sensitivity to unpleasant stimuli in trauma-exposed adults, thus predicting a decrease in LPP difference wave amplitudes for unpleasant images only. (4) Constraint would moderate the relation between childhood trauma and brain activity by increasing discrimination between affective and neutral stimuli in trauma-exposed adults, and therefore predicting an increase in EPN and LPP difference wave amplitudes for both pleasant and unpleasant images.

## Method

### Participants

A sample of *N* = 127 participants were recruited to complete the study. A sample of *N* = 58 actually completed the study. This was attributed to: (1) High attrition, with *n* = 36 participants who completed part one of the experiment but who either did not schedule or did not attend part two. (2) Researchers were unable to use data from *n* = 28 participants due to issues with technology or incomplete data. Of the *n* = 63 EEG data files we did collect from the original sample of *N* = 127, we were able to successfully extract ERPs from *n* = 58 participants.

Thus, participants in this final sample were *N* = 58 (*n* = 43 female, 74.1%) university students in the United States between the ages of 18 and 41 (*M* = 20.53). Participants racially or ethnically identified as: *n* = 34 (58.6%) White/European American, *n* = 19 (32.8%) Black/African American, *n* = 2 (3.4%) Asian, *n* = 2 (3.4%) multiracial, and *n* = 1 (1.7%) did not respond. Finally, *n* = 9 (15.5%) identified as having a Hispanic/Latinx ethnic background. All participants self-reported normal or corrected-to-normal vision. Participants were surveyed for substance use and psychological disorders, with 87.9% of the sample (*n* = 51) reporting no diagnosis. All participants provided informed consent prior to participating in the study and were thoroughly debriefed upon completion. The study was approved by the university’s institutional review board, which uses the Code of Federal Regulations and the Belmont Report instead of the Helsinki declaration.

### Materials

#### Stimuli

We displayed images from the International Affective Pictures System (IAPS; Lang et al., 1997) on an LED computer monitor. Block 1 consisted of neutral images to establish a baseline level of brain activity, and blocks 2 and 3 consisted of either pleasant or unpleasant images. Neutral images included objects, scenes with people, and scenes without people. Pleasant images included affiliative, exciting, and erotic content. Unpleasant images included threatening, disgusting, and mutilative content. Each block consisted of 15 images from each of their three respective categories, amounting to a total of 45 images per block. These 45 images were presented twice for each block, amounting to a total of 90 image presentations per block and 270 image presentations per participant.

#### Hardware

EEG was recorded using six BIOPAC (RRID:SCR_014829) EEG 100 C amplifiers and six pure tin recording electrodes recessed in the fabric of an elastic electrode cap (ElectroCap International). The EEG 10–20 electrode placement system was used. Recording electrodes were located over occipital and centroparietal sites (i.e., O1, O2, P3, Pz, P4, & Cz) with the ground electrode located anterior to Fz; the reference electrode was clipped to the left ear lobe. Impedances did not exceed 10 kOhms of resistance. We recorded data with an online 0.1 Hz high-pass single-pole roll-off filter and 35 Hz low-pass notch filter (60 Hz) sampled at 250 Hz. We recorded vertical electrooculogram (VEOG) using a BIOPAC (RRID:SCR_014829) EOG 100 C amplifier with electrodes placed above and below the left eye.

#### Software

Cedrus SuperLab 4.5 was used to create the experiment and BIOPAC AcqKnowledge 4.1 (RRID:SCR_014829) to acquire the EEG and EOG and preprocess the recorded EEG data. We used EEGLab 12 (RRID:SCR_007292) (Delorme & Makeig, 2004) and ERPLab 5 (RRID:SCR_009574) (Lopez-Calderon & Luck, 2014) with MATLAB to extract, process, and analyze the ERPs offline.

### Measures

#### Adverse Childhood Experience (ACE) Questionnaire

The ACE questionnaire is a 10-item, yes/no survey designed to assess the breadth of traumatic childhood experiences (Felitti et al., 1998). Categories of childhood trauma exposure include abuse and household dysfunction, which are respectively divided into the subcategories: psychological, physical, and sexual abuse; and substance abuse, mental illness, and violence toward mother. An example of a survey item from the psychological abuse category includes, “While you were growing up, during your first 18 years of life, did a parent or other adult in the household often or very often swear at, insult, or put you down?” and from the substance abuse category, “Did you live with anyone who was a problem drinker or alcoholic?” ACE scores are derived by summing up “yes” responses for each and can range from 0 to 10. Of the *N* = 58 college students participating, *n* = 41 had lower ACE scores (i.e., 0–3), and *n* = 17 had higher ACE scores (i.e., 4 or more). The average ACE score was 2.19 with a range of 0 to 8. 29.35% of our sample had 4 or more ACEs. In comparison, according to the Center for Disease Control and Prevention (CDC, 2023), “Nearly two thirds of U.S. adults (63.9%) experienced one or more ACE: 23.1% reported one; 23.5% reported two to three; and 17.3% reported four or more ACEs.”

#### Multidimensional Personality Questionnaire Brief Form (MPQ-BF)

The MPQ-BF is designed to assess emotional coping styles, and it has been used in past research to predict brain activity in response to affective images (Patrick et al., 2002). It is a 155-item, true/false questionnaire that is used to measure *positive emotionality* (i.e., one’s tendency toward positive emotions), *negative emotionality* (i.e., one’s tendency toward negative emotions), and constraint, an index of impulsivity and behavioral control (Patrick et al., 2002). In the present study, we only used the constraint subscale, which included items like, “Often when I get angry I am ready to hit someone.” The constraint dimension was designed to mirror Zuckerman’s (1991) description of *impulsive unsocialized sensation seeking*, a characteristic seen in desensitized individuals with reduced brain arousal and decreased conditionability. Constraint scores are obtained by summing up “yes” responses from the Control, Harm Avoidance, and Traditionalism clusters on the MPQ and can range from 0 to 37. Reliability is good with Chronbach’s alpha coefficients ranging from 0.75 to 0.84 for the primary characteristic scales (Patrick et al., 2002). We used the constraint dimension as a measure of resilience in the present study, with higher scores indicating greater resilience.

#### Emotional Regulation Questionnaire (ERQ)

The ERQ is a 10-item questionnaire designed to measure participants’ inclination toward the emotion regulation strategies of reappraisal and expressive suppression (Gross & John, 2003). Responses to statements like, “When I want to feel more *positive* emotions (such as joy or amusement), I *change what I’m thinking about*,” and “I control my emotions by *not expressing them*,” are indicated on a 7-point Likert scale ranging from 1, “strongly disagree,” to 7, “strongly agree.” Prior research has indicated that internal consistency reliability for cognitive reappraisal (*α =* 0.89-0.90) and expressive suppression (*α =* 0.76-0.80) is good to excellent (Preece et al., 2020). In our sample, internal consistency reliability for our variable of interest, cognitive reappraisal, was good (*α =* 0.83). The cognitive reappraisal dimension was used as a measure of resilience in the present study.

### Procedure

In part one, participants arrived at a designated research lab and completed the survey packet. Researchers debriefed participants at the conclusion of part one by explaining broadly that their participation would help researchers gain a better understanding of emotion processing. However, at this point, researchers did not disclose any other information regarding childhood trauma or resilient characteristics to avoid compromising the blind nature of the study. To incentivize follow-up, researchers also informed participants that a selection of snacks would be provided in part two of the experiment.

In part two of the experiment, researchers collected EEG data from participants who passively viewed IAPS images on a computer monitor 75 cm away while sitting alone in a dimly lit and quiet room. Each stimulus was presented for 1500 ms, with an inter-stimulus interval of 1800–2200 ms and stimulus onset asynchrony of 2000 ms. After reading scripted instructions to participants, we presented block one–neutral images (i.e., objects, scenes with people, and scenes without people)--to establish baseline EEG activity. Blocks two and three–pleasant and unpleasant–were subsequently presented. Pleasant images were presented as block two to half of the participants, and unpleasant to the other half. Participants were given the opportunity to take breaks in between each block. Once participants viewed all three blocks, researchers debriefed and dismissed them.

### ERP Extraction

The continuous EEG for each participant was corrected for eye-blink artifacts using the EOG artifact removal tool, an independent component analysis technique in the AcqKnowledge software (RRID:SCR_014279). Then EEGLab (RRID:SCR_007292) and ERPLab (RRID:SCR_009574) software were used to epoch the continuous EEG from 200 ms pre-stimulus to 1500 ms post-stimulus. To correct for baseline, we subtracted the average value of the pre-stimulus interval from each point in the ERP waveform. We excluded from further analysis any epochs that contained voltage artifacts (amplitudes exceeding ± 100 µV) at any of the six recording electrode sites. Artifact-free epochs were averaged for the ERP analyses, and only participants with less than 25% rejected epochs in any block (neutral, pleasant, unpleasant) were included in our final sample (*N* = 58).

**EPN.** We averaged together the ERP waveforms at the O1 and O2 electrode sites for each block of IAPS images. We used Weinberg et al.’s (2015) time window for the EPN of 175–275 ms post-stimulus. The neutral waveform was subtracted from both the pleasant and unpleasant waveforms to construct difference waves. The negative signed area amplitude (µV x ms) for each difference wave was calculated within the time window using the ERPLab (RRID:SCR_009574) measurement tool.

**LPP.** We averaged together the EEG waveforms at the Pz, P3, P4, and Cz electrode sites for each block of IAPS images. Weinberg et al. (2015) used four post-stimulus time windows for the LPP (i.e., 300–600 ms, 600–1000 ms, 1000–2000 ms, and 2000–3000 ms) to gain a better understanding of the time course of emotion processing. We chose to use their time window of 300–600 ms post-stimulus for the LPP, as this time window was comparable to those used in other previous studies of the LPP (e.g., Pincham et al., 2016; Pollack et al., 1997; Pollack et al., 2001; Shackman et al., 2007). The neutral waveform was subtracted from both the pleasant and unpleasant waveforms to construct difference waves. The positive signed area amplitude (µV x ms) for each difference wave was calculated within the time window using the ERPLab (RRID:SCR_009574) measurement tool.

## Results

**Fig. 1:**
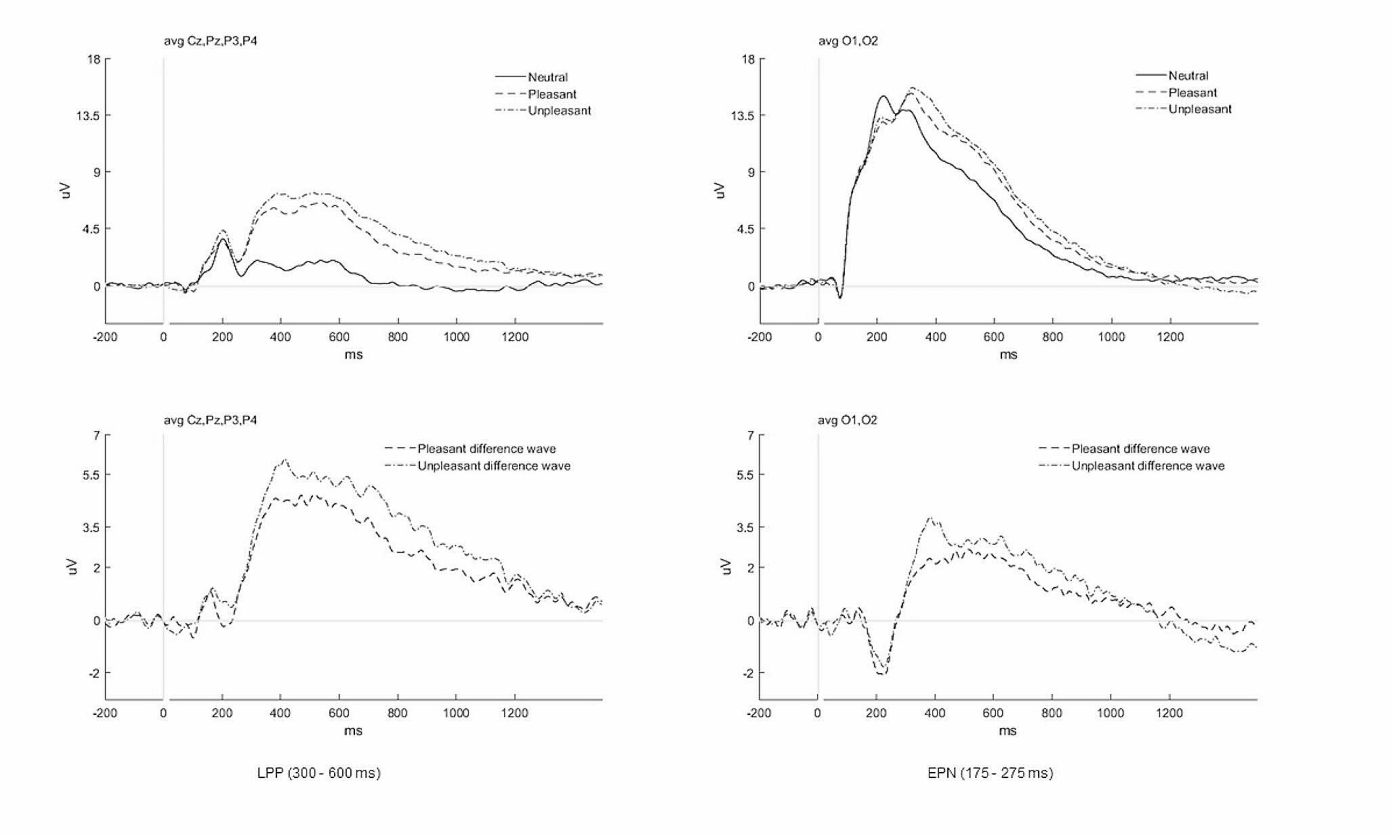
Top panel shows grand average waveforms (*N* = 58) for neutral, pleasant, and unpleasant images. Bottom panel shows grand average difference waveforms (*N* = 58) for pleasant and unpleasant images

Prior to conducting analyses, we confirmed that assumptions of normality were met. Descriptive statistics are shown in Table 1. The statistical tests we performed are described for each hypothesis below, correlations are reported in Table 2, and regression analyses with significant results are displayed in Tables 3 and 4. Grand average ERPs are shown in Fig. 1.

**Table 1:** Descriptive statistics for ACEs, constraint, reappraisal, EPN pleasant difference wave amplitude, EPN unpleasant difference wave amplitude, LPP pleasant difference wave amplitude, and LPP unpleasant difference wave amplitude

									Skewness	
	N		M		SD		Minimum	Maximum	Statistic	Std. Error
ACEs		58		2.19		2.46	0	8	0.98	0.31
Constraint		58		23.59		4.76	12	31	− 0.30	0.31
Reappraisal		58		5.22		1.11	2.33	7.00	− 0.24	0.31
EPN Pleasant Difference Wave		58		0.19		0.19	0	0.77	1.44	0.31
EPN Unpleasant Difference Wave		58		0.20		0.27	0	1.04	1.66	0.31
LPP Pleasant Difference Wave		58		1.36		0.88	0.02	4.18	0.64	0.31
LPP Unpleasant Difference Wave		58		1.59		0.90	0.01	4.42	0.48	0.31

### Correlational Findings

From our exploratory correlational analyses, we discovered a moderate, negative correlation between ACE scores and constraint, which retained its significance even after Bonferroni correction, indicating some association between childhood trauma and a lack of impulse control or a tendency to take risks and engage in unconventional behavior (*r* = − .37, *p* = .004). A few other significant correlations were found, but no longer met the threshold for significance (ɑ = 0.007) after Bonferroni correction was performed, and therefore can only support tentative conclusions. The findings that no longer met the threshold for significance include: (1) A moderate, positive association between constraint and LPP difference wave amplitudes in response to pleasant images (*r* = .32, *p* = .015). This provided preliminary evidence that, consistent with our hypothesis #2, constraint is associated with increased affective discrimination. (2) Finally, we discovered a weak, negative association between reappraisal and LPP difference wave amplitudes in response to unpleasant images (*r* = − .28, *p* = .031). This provided preliminary evidence for our hypothesis #1 that cognitive reappraisal predicts decreased LPP difference wave amplitudes in response to unpleasant images, which would suggest that cognitive reappraisal is associated with reduced reactivity to affective stimuli.

#### Correlation between Childhood Trauma and Brain Activity

There was a weak, negative association between childhood trauma as measured by ACE scores and LPP difference wave amplitudes in response to unpleasant images (*r* = − .29, *p* = .028; Table 2). This suggests that individuals with a history of childhood trauma are more likely to exhibit impaired affective discrimination or desensitization to affective stimuli in later stages of emotion processing. However, we did not find significant correlations for the EPN nor LPP pleasant stimuli. We therefore did not proceed with any further EPN hypothesis testing, in particular.

**Table 2:** Correlations between ACE scores, Constraint, Reappraisal, and difference wave amplitudes

Variable	1	2	3	4	5	6	7
1. ACE							
2. Constraint	− 0.37*						
3. Reappraisal	− 0.16	0.08					
4. Pleasant EPN	− 0.17	− 0.09	0.26				
5. Unpleasant EPN	− 0.16	0.26	0.16	0.33			
6. Pleasant LPP	− 0.23	0.32	− 0.22	− 0.31	0.22		
7. Unpleasant LPP	− 0.29	0.17	− 0.28	− 0.03	− 0.05	0.49*	

*Note* *p < .007 (Bonferroni correction)

### Hierarchical Linear Regression Analyses

We tested our moderation hypotheses using hierarchical linear regression analyses, with ACE scores as the independent variable, constraint or cognitive reappraisal as control variables, and signed area amplitudes of the LPP difference waves as the dependent variable. For each analysis, we selected one of the two dependent variables (i.e., pleasant LPP and unpleasant LPP), then entered the control variable of interest (i.e., constraint or reappraisal) in step one, the predictor variable (i.e., ACE Scores) in step two; and finally the interaction variable in step three. We applied the Bonferroni correction to each hierarchical linear regression analysis to control the familywise error rate, which resulted in a significance level of α = 0.017 for each test.

#### Constraint as a Moderator of the Relation between Childhood Trauma and Emotion Processing

We conducted a hierarchical linear regression analysis to test our hypothesis that constraint moderates the relation between childhood trauma and emotion processing of unpleasant images. The model significantly improved when we added the interaction term in the third step, indicating that two variables explain 19% of the variance in the LPP difference wave amplitudes in response to unpleasant images (*R*^*2*^ = 0.19, *F*(2, 56) = 6.62, *p* = .013). Particularly, ACE scores had a main effect on LPP difference wave amplitudes in response to unpleasant images (*β* = -2.11, *p* = .006; Table 3) and constraint moderated the relation between childhood trauma and affective discrimination between neutral and unpleasant images (*β* = 1.79, *p* = .013; Table 3).

In another hierarchical linear regression analysis, constraint independently predicted emotion processing of pleasant images but did not moderate the relation between childhood trauma and emotion processing of pleasant images. We found that constraint significantly improved the model in step one, explaining 10% of the variance in LPP difference wave amplitudes in response to pleasant images (*R*^*2*^ = 0.10, *F*(2, 56) = 6.25, *p* = .015). Constraint had a main effect on LPP difference wave amplitudes in response to pleasant images (*β* = 0.32, *p* = .015, Table 3).

These results partially support our hypothesis that constraint moderates the relation between childhood trauma and emotion processing; while the majority of participants with childhood trauma displayed reduced sensitivity to unpleasant images, those with high levels of constraint were more sensitive to unpleasant images, similar to controls. Further, we found that constraint independently predicted enhanced LPP difference wave amplitudes in response to pleasant images, indicating increased positive affect in response to these stimuli as compared to those with lower levels of constraint. However, these results only partially support our hypotheses because significance was not consistently found for both pleasant and unpleasant images.

**Table 3:** Regression for Pleasant and Unpleasant LPP difference waves with Constraint as control variable

			Unstandardized Coefficients		Standardized Coefficients					
Step	Outcome Variable	Predictor	*B*	*SE*	*β*	*p*	*R* ^*2*^	*R* ^*2*^ *change*	*F*	*p*
Regression Analysis of ACE Score, Constraint on Pleasant LPP Difference Wave Amplitudes										
1	Pleasant LPP Difference Wave	Constraint	0.06	0.02	0.32	0.015*	0.10	0.10	6.25	0.015*
2	Pleasant LPP Difference Wave	Constraint ACE Score	0.05 − 0.05	0.03 0.05	0.27 − 0.13	0.055 0.345	0.12	0.02	0.91	0.345
3	Pleasant LPP Difference Wave	Constraint ACE Score Constraint x ACE Score	0.05 0.002 − 0.002	0.03 0.27 0.01	0.29 0.01 − 0.13	0.107 0.993 0.856	0.12	0.001	0.03	0.856
Step	Outcome Variable	Predictor	*B*	*SE*	*β*	*p*	*R* ^*2*^	*R* ^*2*^ *change*	*F*	*p*
Regression Analysis of ACE Score, Constraint on Unpleasant LPP Difference Wave Amplitudes										
1	Unpleasant LPP Difference Wave	Constraint	0.03	0.03	0.17	0.205	0.03	0.03	1.64	0.205
2	Unpleasant LPP Difference Wave	Constraint ACE Score	0.01 − 0.10	0.03 0.05	0.07 − 0.26	0.610 0.064	0.09	0.06	3.58	0.064
3	Unpleasant LPP Difference Wave	Constraint ACE Score Constraint x ACE Score	− 0.04 − 0.77 0.03	0.03 0.27 0.01	− 0.20 -2.11 1.79	0.246 0.006* 0.013*	0.19	0.10	6.62	0.013*

*Note* *p < .017 (Bonferroni correction)

#### The Relation between Cognitive Reappraisal, Childhood Trauma, and Brain Activity

To directly test our hypothesis that cognitive reappraisal and childhood trauma predict decreased reactivity to unpleasant images and decreased affective discrimination, we performed a hierarchical linear regression. The results of the regression indicated that two predictors explained 20% of the variance in LPP difference wave amplitudes (*R*^*2*^ = 0.20, *F*(2, 56) = 7.87, *p* = .007). In particular, ACE scores were significant predictors of LPP difference wave amplitudes in response to unpleasant images (*β* = − 0.34, *p* = .007; Table 4). Cognitive reappraisal was also a significant predictor of LPP difference wave amplitudes in response to unpleasant images (*β* = − 0.34, *p* = .008; Table 4). However, the interaction term was not a significant predictor of LPP difference wave amplitudes, suggesting that cognitive reappraisal does not moderate the relation between childhood trauma and brain activity as predicted in our hypothesis #3.

We also conducted a hierarchical linear regression for LPP difference wave amplitudes in response to pleasant images. We found that two variables explained 12% of the variance in LPP difference wave amplitudes in response to pleasant images (*R*^*2*^ = 0.12, *F*(2, 56) = 4.54, *p* = .038; Table 4). Specifically, both cognitive reappraisal (*β* = − 0.27, *p* = .043) and ACE scores (*β* = − 0.27, *p* = .038) had a main effect on LPP difference waves in response to pleasant images (Table 4). However, after applying the Bonferroni correction, these results no longer met the threshold for significance (i.e., α = 0.017), and can therefore only support tentative conclusions.

These results are consistent with our hypothesis #1 that cognitive reappraisal predicts reduced reactivity to unpleasant stimuli in the later stages of emotion processing (i.e., in the time window of the LPP).

**Table 4:** Regression for Pleasant and Unpleasant LPP Difference Waves with Reappraisal as control variable

			Unstandardized Coefficients		Standardized Coefficients					
Step	Outcome Variable	Predictor	*B*	*SE*	*β*	*p*	*R* ^*2*^	*R* ^*2*^ *change*	*F*	*p*
Regression Analysis of ACE Score, Reappraisal on Pleasant LPP Difference Wave Amplitudes										
1	Pleasant LPP Difference Wave	Reappraisal	− 0.18	0.10	− 0.22	0.095	0.05	0.05	2.89	0.095
2	Pleasant LPP Difference Wave	Reappraisal ACE Score	− 0.21 − 0.10	0.10 0.05	− 0.27 − 0.27	0.043 0.038	0.12	0.07	4.54	0.038
3	Pleasant LPP Difference Wave	Reappraisal ACE Score Reappraisal x ACE Score	− 0.19 − 0.06 − 0.01	0.14 0.25 0.05	− 0.25 − 0.16 − 0.12	0.168 0.824 0.865	0.12	0	0.03	0.865
Step	Outcome Variable	Predictor	*B*	*SE*	*β*	*p*	*R* ^*2*^	*R* ^*2*^ *change*	*F*	*p*
Regression Analysis of ACE Score, Reappraisal on Unpleasant LPP Difference Wave Amplitudes										
1	Unpleasant LPP Difference Wave	Reappraisal	− 0.23	0.10	− 0.28	0.031	0.08	0.08	4.89	0.031
2	Unpleasant LPP Difference Wave	Reappraisal ACE Score	− 0.28 − 0.13	0.10 0.05	− 0.34 − 0.34	0.008* 0.007*	0.20	0.12	7.87	0.007*
3	Unpleasant LPP Difference Wave	Reappraisal ACE Score Reappraisal x ACE Score	− 0.20 0.06 − 0.04	0.14 0.25 0.05	− 0.25 0.17 − 0.52	0.140 0.799 0.435	0.21	0.01	0.62	0.435

*Note* *p < .017 (Bonferroni correction)

#### Summary of Results

In sum, our results supported the following hypotheses: (1) Cognitive reappraisal predicted decreased sensitivity to unpleasant images in the later stages of emotion processing, and therefore smaller LPP difference wave amplitudes in response to unpleasant images, specifically. (2) Constraint predicted increased affective discrimination as evidenced by larger LPP difference wave amplitudes between neutral and pleasant images. (4) Constraint moderated the relation between childhood trauma and brain activity by predicting enhanced discrimination between unpleasant and neutral stimuli in trauma-exposed adults, as measured by LPP difference wave amplitudes. These hypotheses were only partially supported because significant results were not consistently found for the hypothesized image sets (i.e., pleasant and/or unpleasant).

We did not find support for the following hypothesis: (3) Cognitive reappraisal moderates the relation between childhood trauma and emotion processing in trauma-exposed adults.

## Discussion

The present study examined the association between childhood trauma, resilience, and ERP correlates of emotion processing. Consistent with our hypothesis, we found that as childhood trauma (i.e., ACE scores) increased, constraint or impulse control decreased. Individuals with high levels of childhood trauma were also less sensitive to emotional images and less adept at differentiating them from neutral images. Van Bodegom et al. (2017) suggest that this may be due to chronically elevated levels of cortisol, which contributes to downregulation of corticosteroid receptors, desensitization to stress hormones, and reduced negative feedback to the hypothalamic-pituitary-adrenal (HPA) axis (i.e., neuroendocrine system that regulates stress). Due to this suppressed negative feedback, individuals with childhood trauma often display hyper-reactivity of the HPA axis to external stressors (van Bodegom et al., 2017). Behaviorally, neurodevelopmental differences resulting from childhood trauma may present as anhedonia or reduced pleasure response, decreased sensitivity to unpleasant stimuli, impulsivity, among other outcomes.

ERP research has shown similar results; individuals with a history of childhood trauma tend to display impaired affective discrimination as evidenced by generally reduced ERP amplitudes in response to emotional versus neutral stimuli as compared to controls, with angry faces being the exception (e.g., Chu et al., 2016; Cicchetti & Curtis, 2005; Curtis & Cicchetti, 2011; Pollack et al., 1997; Pollack et al., 2001; Shackman et al., 2007). However, the association between ACEs and difference wave amplitudes was not found for EPN difference waves in our study, which may be due to our relatively small sample size. Sill et al. (2020), for example, did find this negative, albeit small, association between ACEs and the EPN difference wave amplitude for both pleasant and unpleasant images in a sample of 90 adolescents. In sum, we found results consistent with an existing body of evidence showing that childhood trauma may desensitize individuals to emotional images or impair one’s ability to differentiate emotional and neutral images. In addition, we found that resilient characteristics such as constraint and cognitive reappraisal are associated with emotion processing: Constraint predicted increased sensitivity to pleasant stimuli and cognitive reappraisal predicted decreased sensitivity to unpleasant stimuli.

Research on resilient characteristics in relation to ERPs has shown that high scores on impulse control or constraint tend to predict increased mean LPP amplitudes in response to emotional images (Munk et al., 2020), whereas high scores on cognitive reappraisal tend to predict decreased reactivity to unpleasant images, and thus reduced mean LPP amplitudes in response to unpleasant or aversive images (Harrison & Chassy, 2019). Pincham et al. (2016) found that at-risk adolescents initially showed decreased affective discrimination, as evidenced by reduced mean LPP amplitudes, but after nine months of extensive therapy aimed at improving emotional coping strategies and bolstering resilient characteristics, these amplitudes increased to match that of controls, demonstrating improved sensitivity to emotional stimuli. In the present study, we found results similar to Pincham et al.’s (2016) study. Individuals with a history of childhood trauma displayed reduced unpleasant LPP difference wave amplitudes as compared to individuals with no history of childhood trauma, but LPP difference wave amplitudes in response to pleasant images tended to increase with higher self-reported constraint. Cognitive reappraisal, on the other hand, was associated with reduced LPP difference wave amplitudes in response to unpleasant images, consistent with prior research (e.g., Cauwenberge et al., 2017; Harrison & Chassy, 2019; Langeslag & Strien, 2018). Thus, constraint may be particularly useful for predicting positive emotions, while cognitive reappraisal may be particularly useful for predicting negative emotions.

While cognitive reappraisal is a teachable emotion processing technique that supports resilience, future research may benefit from investigating whether constraint or impulse control can also be taught in a similar manner. Korponay et al. (2019) found that a mindfulness meditation intervention did *not* result in improved impulse control or constraint, as measured by performance on a go/no-go task. This provides preliminary evidence that constraint or impulse control may be a more fixed resilient characteristic, but further research is needed to determine if there are therapeutic interventions that effectively improve constraint. Neuroimaging could also be used to track changes in the structure and function of brain regions supporting impulse control and controlled, top-down emotion processing, such as the prefrontal cortex, with various therapeutic interventions over time.

In a systematic review of neuroimaging findings among individuals with childhood trauma, Cassiers et al. (2018) found that the most common brain abnormality across all subtypes of childhood trauma (e.g., emotional, physical, and sexual maltreatment) was reduced frontal cortical volume. Poor top-down control of emotions due to structural and functional deficits in the prefrontal cortex may serve as a plausible explanation for the reduced constraint or impulse control associated with childhood trauma in the present study, and reduced ERP amplitudes are typically associated with externalizing behaviors (Chu et al., 2016). While Cassiers et al. (2018) found other brain abnormalities in structure and function within each subtype of childhood trauma, the associations were complex and inconsistent across childhood trauma subtypes. Thus, further research would benefit from delineating neural correlates of each subtype of trauma, rather than neural correlates of childhood trauma more broadly. This is important for identifying neural mediators between childhood trauma and psychopathology.

### Constraint

Prior research has shown reduced brain volume and density among people with childhood trauma in regions that are implicated in impulse control, constraint, working memory, and cross-regional connectivity, such as the prefrontal cortex, orbitofrontal cortex, hippocampus, and corpus callosum (Cohen et al., 2006; De Bellis et al., 2002, 2015; De Brito et al., 2013; Teicher et al., 2003, 2004; Vythilingam et al., 2002). Hallowell et al. (2019) found that adults with childhood trauma performed worse on a verbal working memory task than controls, and that neural activity mediated the relation between childhood trauma and impulsivity or constraint in these individuals. Various mediators between childhood trauma and brain development have been proposed, such as increased cortisol and accelerated neuronal pruning due to chronic stress in the critical period of brain development, and thus stunted development of frontal regions (Jeong et al., 2021).

In the present study, we found that individuals with childhood trauma were significantly more likely to self-report low levels of constraint and inhibition, with greater engagement in risk-taking and unconventional behaviors. We were able to use self-reported levels of constraint to predict brain activity in response to affective images. Specifically, higher levels of constraint independently predicted increased sensitivity to pleasant images as measured via LPP difference wave amplitudes. It also moderated the relation between childhood trauma and unpleasant LPP difference wave amplitudes; while childhood trauma predicted reduced sensitivity to unpleasant images, those with childhood trauma who were also high in constraint were more sensitive to unpleasant images, as shown by increased LPP difference wave amplitudes. Therapeutic intervention aimed at improving emotional coping strategies in adolescents with childhood trauma seems to improve affective discrimination over time (Pincham et al., 2016), but the specific emotional coping strategies taught in these interventions are often poorly measured or defined, thus making it difficult to infer causal relations between specific resilient characteristics and indices of emotion processing. Future research should investigate whether constraint is an emotion processing technique that can be taught and potentially contribute to positive brain changes and improved resilience.

### Cognitive Reappraisal

In the present study, cognitive reappraisal seemed to predict brain activity independent from childhood trauma, whereas constraint interacted with childhood trauma to predict brain activity. Our data suggest that cognitive reappraisal predicts reduced reactivity to unpleasant stimuli in later stages of emotion processing. In other words, cognitive reappraisal may predict reduced LPP amplitudes in response to unpleasant images (i.e., thus producing a smaller amplitude difference wave).

Our data are consistent with prior research in healthy individuals showing that higher cognitive reappraisal scores predicted reduced LPP amplitudes in response to unpleasant images (e.g., Harrison & Chassy, 2019; Langeslag & Strien, 2018; Munk et al., 2020; van Cauwenberge et al., 2017). Thus, emotion regulation strategies such as cognitive reappraisal may function in the later stages of emotion processing but not necessarily in the earlier stages. Our data suggest that cognitive reappraisal is associated with reduced sensitivity to unpleasant stimuli. Chen et al. (2020) found support for this, such that cognitive reappraisal-related emotion processing techniques decreased LPP amplitudes in response to unpleasant stimuli 300-1,700 ms post-stimulus onset. However, Liu et al. (2019) also found that cognitive reappraisal can be used to upregulate positive emotions in response to pleasant stimuli. Notably, the children in the study were explicitly asked to reappraise the meaning of pleasant images. Thus, cognitive reappraisal’s impact on positive emotions may be more explicit than implicit, but further research is needed to support this assumption.

### Theoretical Implications

Decreased sensitivity to affective stimuli often seen in individuals with childhood trauma may function to conserve energy in environments where stressors are common, and thus may actually be helpful in traumatic environments. Characteristics such as constraint and cognitive reappraisal could be recruited in novel environments to increase positive affect or decrease negative affect and aid in adaptive emotion processing in novel environments. This is important to note when considering interventions for individuals with childhood trauma. One should ask: Would this individual reap more immediate benefits from a change in their home environment or therapeutic intervention? Teaching resilient characteristics such as constraint and reappraisal may be more beneficial for adults who are no longer immersed in the traumatic environments of their childhood.

The theory of constructed emotion posits that past experiences are used to construct an internal model of the world that predicts outcomes of sensory environments (Barrett, 2017). We propose that characteristics or techniques such as cognitive reappraisal and constraint function to improve emotion processing by updating one’s internal model of the world irrespective of past experience. Reappraisal, for example, is the practice of positively reframing certain stimuli or circumstances. This encourages one to selectively update one’s internal model so that it predicts more positive outcomes, despite a large repertoire of negative past experiences in individuals with childhood trauma. Constraint, on the other hand, is often described as impulse control and avoidance of risk or unconventional behavior. Constraint may thus drive one to pause in novel situations and consider whether a strong emotional response is necessary. These techniques are powerful in that they may update the internal model of an individual with a history of childhood trauma separate from traumatic experiences in childhood.

### Limitations and Future Directions

Past research has shown that both pleasant and unpleasant IAPS images evoke strong LPP responses compared with neutral images (Dolcos & Cabeza, 2002). Thus, we may have seen more significant, but perhaps weaker results for pleasant images if our sample size had been larger. The same could be said in regard to our results for the EPN component, which may have been prone to type II error due to our limited sample size. Sill et al. (2020), for example, used IAPS images and found significantly reduced EPN difference wave amplitudes for both pleasant and unpleasant images in 90 participants with a history of childhood trauma. Our results suggest that the influence of childhood trauma and resilient characteristics may be clearer in later stages of emotion processing. While childhood trauma was associated with reduced sensitivity to emotional images across all image sets and ERP components in the present study, childhood trauma was more strongly associated with reduced sensitivity to emotional images in later stages of emotion processing (i.e., for the LPP component), and significance was only detected for the LPP component in the present study.

The quality of our EEG data may be limited due to the small number of recording electrodes used(i.e., six). With the BIOPAC system, each recording electrode requires its own amplifier, and we were only able to acquire six amplifiers for our lab. Future studies should aim to replicate our results with additional electrodes.

Future research should be careful to delineate trauma subtypes in order to disentangle conflicting findings on neural correlates of childhood trauma, as brain imaging studies have shown differential activity of brain regions dependent on the type of childhood trauma. Puetz et al. (2020) found that childhood abuse was associated with hyper-activation of the ventral amygdala in response to threatening stimuli, childhood neglect was associated with hyper-activation of a fronto-parietal network involved in social and cognitive processing, and, interestingly, combined childhood abuse and neglect was associated with *hypo*-activation of both regions. Hypo-activation of the ventromedial prefrontal cortex (vmPFC) and hyper-activation of the amygdala is a pattern commonly seen in individuals with post-traumatic stress disorder (PTSD) (Killgore et al., 2014), and PTSD symptom severity is associated with the extent of childhood trauma (Yehuda et al., 2001). In a systematic review, Eaton et al. (2022) found that resilient individuals showed greater frontal activation associated with increased top-down control of emotions, greater basal ganglia activation associated with increased reward sensitivity, and reduced amygdala activation associated with decreased threat response, as well as increased grey matter volume in the frontal regions and hippocampus of the brain. When considered in the context of the present study, these neuroimaging studies help clarify seemingly contradictory ERP patterns in resilient individuals. For example, in the present study, reappraisal was associated with reduced LPP amplitudes in response to unpleasant images, but constraint was associated with increased LPP amplitudes in response to pleasant images. The former result may stem from decreased amygdala activation, while the latter result may stem from increased basal ganglia activation, as seen in the resilient individuals in Eaton et al.’s (2022) study.

While childhood trauma predicts psychopathology, there are many people with childhood trauma who are resilient and do not develop mental illnesses. Even among those who do, there are discrepant findings on the association between mental illnesses and ERP responses to emotional images, as the magnitude of the amplitude seems to be moderated by such things as differences in PTSD and anxiety symptomatology (e.g., Cicchetti & Curtis, 2005; Klimova et al., 2013; Lanius et al., 2010). Our main conclusion is that adults with a history of childhood trauma may have trouble *differentiating* between affective and neutral stimuli. One behavioral manifestation of reduced affective discrimination in these individuals may be anhedonia and a reduced pleasure response. This may contribute to a greater risk for depressed mood in times of stress, as these individuals may be less likely to seek out positive emotional experiences or do not respond favorably to pleasurable stimuli in general (Heo et al., 2021). It may be conceptualized as a manifestation of the exhaustion stage in the GAS model, such that the body no longer has the resources to enjoy pleasant stimuli to the same extent as it did prior to chronic stress (Selye, 1936). Behavioral correlates of ERPs may be further elucidated via experimental paradigms requiring participants to engage in psychological or cognitive tasks, such as go/no-go or oddball tasks, during both EEG and fMRI scans. This would allow us to better compare results across neuroimaging techniques and behavioral tasks, thus enhancing our understanding of the relations between brain activity and behavior.

## Conclusion

The present study analyzed the association between childhood trauma, resilience, and ERP correlates of emotion processing. Our findings provide promising evidence that one can utilize certain emotion regulation strategies to protect against the deleterious effects of childhood trauma on emotion processing. More specifically, the present study provides evidence that specific emotion regulation strategies may differentially predict brain activity, with constraint serving as a moderator and predicting increased positive emotion, and cognitive reappraisal having a main effect and predicting decreased negative emotions. Future research would benefit from experimental paradigms to home in on causality, complementary neuroimaging data to identify neural substrates of emotion regulation, and a greater effort to delineate neural correlates of specific trauma subtypes. Ultimately, the purpose of this body of research is to improve the lives of individuals affected by childhood trauma; identifying specific, teachable resilient characteristics and protective factors through research is one step closer to achieving this goal.

## References

[CR1] Barrett, L. F. (2017). The theory of constructed emotion: An active inference account of interoception and categorization. *Social Cognitive and Affective Neuroscience*, *12*(1), 1–23. 10.1093/scan/nsw154. 27798257 10.1093/scan/nsw154 PMC5390700

[CR2] Brennan, G. M., & Baskin-Sommers, A. R. (2018). Brain-behavior relationships in externalizing: P3 amplitude reduction reflects deficient inhibitory control. *Behavior Brain Research*, *337*, 70–79. 10.1016/j.bbr.2017.09.045. 10.1016/j.bbr.2017.09.045 28966148

[CR3] Cassiers, L. L. M., Sabbe, B. G. C., Schmaal, L., Veltman, D. J., Penninx, B. W. J. H., & Eede, F. V. D. (2018). Structural and functional brain abnormalities associated with exposure to different childhood trauma subtypes: A systematic review of neuroimaging findings. *Frontiers in Psychiatry*, *9*(329), 1–17. 10.3389/fpsyt.2018.00329. 30123142 10.3389/fpsyt.2018.00329 PMC6086138

[CR4] Centers for Disease Control and Prevention (2023, June 29). *Prevalence of adverse childhood experiences among U.S. adults - behavioral risk factor surveillance system, 2011–2020*. Centers for Disease Control and Prevention. https://www.cdc.gov/mmwr/volumes/72/wr/mm7226a2.htm.

[CR5] Chen, S., Yu, K., Yang, J., & Yuan, J. (2020). Automatic reappraisal-based implementation intention produces early and sustainable emotion regulation effects: Event-related potential evidence. *Frontiers in Behavioral Neuroscience*, *14*(89), 1–11. 10.3389/fnbeh.2020.00089. 32765230 10.3389/fnbeh.2020.00089 PMC7381194

[CR6] Chu, D. A., Bryant, R. A., Gatt, J. M., & Harris, A. W. F. (2016). Failure to differentiate between threat-related and positive emotion cues in healthy adults with childhood interpersonal or adult trauma. *Journal of Psychiatric Research*, *78*, 31–41. 10.1016/j.jpsychires.2016.03.006. 27055015 10.1016/j.jpsychires.2016.03.006

[CR7] Cicchetti, D., & Curtis, W. J. (2005). An event-related potential study of the processing of affective facial expressions in young children who experienced maltreatment during the first year of life. *Developmental Psychopathology*, *17*, 641–677. 10.1017/s0954579405050315. 10.1017/S0954579405050315 16262986

[CR8] Cohen, R. A., Grieve, S., Hoth, K. F., Paul, R. H., Sweet, L., Tate, D., Gunstad, J., Stroud, L., McCaffery, J., Hitsman, B., Niaura, R., Clark, C. R., MacFarlane, A., Bryant, R., Gordon, E., & Williams, L. M. (2006). Early life stress and morphometry of the adult anterior cingulate cortex and caudate nuclei. *Biological Psychiatry*, *59*, 975–982. 10.2190/5R62-9PQY-0NEL-TLPA. 16616722 10.1016/j.biopsych.2005.12.016

[CR9] Curtis, W. J., & Cicchetti, D. (2011). Affective facial expression processing in young children who have experienced maltreatment during the first year of life: An event-related potential study. *Developmental Psychopathology*, *23*(2), 373–395. 10.1017/s0954579411000125. 10.1017/S0954579411000125 23786684

[CR10] De Bellis, M. D., Keshavan, M. S., Frustaci, K., Shifflett, H., Iyengar, S., Beers, S. R., & Hall, J. (2002). Superior temporal gyrus volumes in maltreated children and adolescents with PTSD. *Biological Psychiatry*, *51*, 544–552. 10.1016/S0006-3223(01)01374-9. 11950456 10.1016/s0006-3223(01)01374-9

[CR11] De Bellis, M. D., Hooper, S. R., Chen, S. D., Provenzale, J. M., Boyd, B. D., Glessner, C. E., MacFall, J. R., Payne, M. E., Rybczynski, R., & Woolley, D. P. (2015). Posterior structural brain volumes differ in maltreated youth with and without chronic posttraumatic stress disorder. *Development and Psychopathology*, *27*, 1555–1576. 10.1017/S0954579415000942. 26535944 10.1017/S0954579415000942 PMC4815906

[CR12] De Brito, S. A., Viding, E., Sebastian, C. L., Kelly, P. A., Mechelli, A., Maris, H., & McCrory, E. J. (2013). Reduced orbitofrontal and temporal grey matter in a community sample of maltreated children. *Journal of Child Psychology and Psychiatry*, *54*, 105–112. 10.1111/j.1469-7610.2012.02597.x. 22880630 10.1111/j.1469-7610.2012.02597.x

[CR13] Denckla, C. A., Cicchetti, D., Kubzansky, L. D., Seedat, S., Teicher, M. H., Williams, D. R., & Koenen, K. C. (2020). Psychological resilience: An update on definitions, a critical appraisal, and research recommendations. *European Journal of Psychotraumatology*, *11*(1), 1–18. 10.1080/20008198.2020.1822064. 10.1080/20008198.2020.1822064 PMC7678676 33244362

[CR14] Dolcos, F., & Cabeza, R. (2002). Event-related potentials of emotional memory: Encoding pleasant, unpleasant, and neutral pictures. *Cognitive Affective & Behavioral Neuroscience*, *2*(3), 252–263. 10.3758/cabn.2.3.252. 10.3758/cabn.2.3.252 12775189

[CR15] Eaton, S., Cornwell, H., Hamilton-Giachritsis, C., & Fairchild, G. (2022). Resilience and young people’s brain structure, function and connectivity: A systematic review. *Neuroscience and Biobehavioral Reviews*, *132*, 936–956. 10.1016/j.neubiorev.2021.11.001. 34740756 10.1016/j.neubiorev.2021.11.001

[CR16] Felitti, V. J., Anda, R. F., Nordenberg, D., Williamson, D. F., Spitz, A. M., Edwards, V., Koss, M. P., & Marks, J. S. (1998). Relationship of childhood abuse and household dysfunction to many of the leading causes of death in adults. *American Journal of Preventive Medicine*, *14*(4), 245–258. 10.1016/s0749-3797(98)00017-8. 9635069 10.1016/s0749-3797(98)00017-8

[CR17] Gluckman, P. D., Hanson, M. A., & Beedle, A. S. (2007). Early life events and their consequences for later disease: A life history and evolutionary perspective. *American Journal of Human Biology*, *19*, 1–19. 10.1002/ajhb.20590. 10.1002/ajhb.20590 17160980

[CR19] Gross, J. J., & John, O. P. (2003). Individual differences in two emotion regulation processes: Implications for affect, relationships, and well-being. *Journal of Personality and Social Psychology*, *85*, 348–362. 10.1037/0022-3514.85.2.348. 12916575 10.1037/0022-3514.85.2.348

[CR20] Hallowell, E. S., Oshri, A., Liebel, S. W., Liu, S., Duda, B., Clark, U. S., & Sweet, L. H. (2019). The mediating role of neural activity on the relationship between childhood maltreatment and impulsivity. *Child Maltreatment*, *24*(4), 1–11. 10.1177/1077559519835975. 10.1177/1077559519835975 PMC6764908 30917694

[CR21] Harrison, N. R., & Chassy, P. (2019). Habitual use of reappraisal to regulate emotions is associated with decreased amplitude of the late positive potential (LPP) elicited by threatening pictures. *Journal of Psychophysiology*, *33*(1), 22–31. 10.1027/0269-8803/a000202.

[CR22] Heo, S., Kwon, Y. J., Lee, H., Lee, H. S., Yoon, H., Shim, S., & Kim, J. S. (2021). Electrophysiological changes related to childhood trauma in patients with major depressive disorder: An event-related potential study. *Clinical Psychopharmacology and Neuroscience*, *20*(1), 167–179. 10.1016/j.jpsychires.2021.05.034. 10.9758/cpn.2022.20.1.167 PMC8813325 35078959

[CR23] Hughes, K., Bellis, M. A., Hardcastle, K. A., Sethi, D., Butchart, A., Mikton, C., Jones, L., & Dunne, M. P. (2017). The effect of multiple adverse childhood experiences on health: A systematic review and meta-analysis. *Lancet Public Health*, *2*, e356–366. 10.1016/s2468-2667(17)30118-4. 29253477 10.1016/S2468-2667(17)30118-4

[CR24] Jackson, M. (2014). Evaluating the role of Hans Selye in the modern history of stress. In D. Cantor, & E. Ramsden (Eds.), *Stress, shock, and adaptation in the twentieth century*. University of Rochester. 26962615

[CR25] Jeong, H. J., Durham, E. L., Moore, T. M., Dupont, R. M., McDowell, M., Cardenas-Iniguez, C., Micciche, E. T., Berman, M. G., Lahey, B. B., & Kaczkurkin, A. N. (2021). The association between latent trauma and brain structure in children. *Translational Psychiatry*, *11*(240), 1–9. 10.1038/s41398-021-01357-z. 33895776 10.1038/s41398-021-01357-z PMC8068725

[CR26] Killgore, W. D. S., Britton, J. C., Schwab, Z. J., Price, L. M., Weiner, M. R., Gold, A. L., Rosso, I. M., Simon, N. M., Pollak, M. H., & Rauch, S. L. (2014). Cortico-limbic responses to masked affective faces across ptsd, panic disorder, and specific phobia. *Depression and Anxiety*, *31*(2), 150–159. 10.1002/da.22156. 23861215 10.1002/da.22156 PMC4593618

[CR27] Klimova, A., Bryant, R. A., Williams, L. M., & Felmingham, K. L. (2013). Dysregulation in cortical reactivity to emotional faces in PTSD patients with high dissociation symptoms. *European Journal of Psychotraumatology*, *4*(10). 10.3402/ejpt.v4i0.20430. 10.3402/ejpt.v4i0.20430 PMC3764312 24020010

[CR28] Korponay, C., Dentico, D., Kral, T. R. A., Ly, M., Kruis, A., Davis, K., Goldman, R., Lutz, A., & Davidson, R. J. (2019). The effect of mindfulness meditation on impulsivity and its neurobiological correlates in healthy adults. *Scientific Reports*, *9*(11963), 1–17. 10.1038/s41598-019-47662-y. 31427669 10.1038/s41598-019-47662-y PMC6700173

[CR29] Lang, P. J., Bradley, M. M., & Cuthbert, B. N. (1997). *International Affective Picture System (IAPS): Technical manual and affective ratings*. NIMH Center for the Study of Emotion and Attention.

[CR30] Langeslag, S. J. E., & Sanchez, M. E. (2018). Down-regulation of love feelings after a romantic break-up: Self-report and electrophysiological data. *Journal of Experimental Psychology*, *147*(5), 720–733. 10.1037/xge0000360. 28857575 10.1037/xge0000360

[CR31] Langeslag, S. J. E., & Strien, J. W. V. (2018). Cognitive reappraisal of snake and spider pictures: An event-related potentials study. *International Journal of Psychophysiology*, *130*, 1–8. 10.1016/j.ijpsycho.2018.05.010. 29859220 10.1016/j.ijpsycho.2018.05.010

[CR32] Lanius, R. A., Vermetten, E., Loewenstein, R. J., Brand, B., Schmahl, C., Bremner, J. D., et al. (2010). Emotion modulation in PTSD: Clinical and neurobiological evidence for a dissociative subtype. *The American Journal of Psychiatry*, *167*, 640–647. https://doi.org/10.1176%2Fappi.ajp.2009.09081168. 20360318 10.1176/appi.ajp.2009.09081168 PMC3226703

[CR33] LaRowe, S. D., Patrick, C. J., Curtin, J. J., & Kline, J. P. (2006). Personality correlates of startle habituation. *Biological Psychology*, *72*(3), 257–264. 10.1016/j.biopsycho.2005.11.008. 16406215 10.1016/j.biopsycho.2005.11.008

[CR34] Lecei, A., & Winkel, R. (2020). Hippocampal pattern separation of emotional information determining risk or resilience in individuals exposed to childhood trauma: Linking exposure to neurodevelopmental alterations and threat anticipation. *Neuroscience and Biobehavioral Reviews*, *108*, 160–170. 10.1016/j.neubiorev.2019.11.010 31743725 10.1016/j.neubiorev.2019.11.010

[CR35] Liu, W., Liu, F., Chen, L., Jiang, Z., & Shang, J. (2019). Cognitive reappraisal in children: Neuropsychological evidence of up-regulating positive emotion from an ERP study. *Frontiers in Psychology*, *10*(147), 1–10. 10.3389/fpsyg.2019.00147. 30853920 10.3389/fpsyg.2019.00147 PMC6396714

[CR36] Luck, S. J. (2012). Event-related potentials. In H. Cooper, P. M. Camic, D. L. Long, A. T. Panter, D. Rindskopf, & K. J. Sher (Eds.), *APA handbooks in psychology®. APA handbook of research methods in psychology, Vol. 1. Foundations, planning, measures, and psychometrics* (pp. 523–546). American Psychological Association. 10.1037/13619-028.

[CR37] MacNamara, A., Joyner, K., & Klawohn, J. (2022). Event-related potential studies of emotion regulation: A review of recent progress and future directions. *International Journal of Psychophysiology*, *176*, 73–88. 10.1016/j.ijpsycho.2022.03.008. 35346736 10.1016/j.ijpsycho.2022.03.008 PMC9081270

[CR38] Munk, A. J. L., Schmidt, N. M., & Hennig, J. (2020). Motivational salience, impulsivity and testosterone in free cycling women: An ERP study. *Personality and Individual Differences*, *160*, 1–7. 10.1016/j.paid.2020.109902.

[CR39] Patrick, C. J., Curtin, J. J., & Tellegen, A. (2002). Development and validation of a brief form of the Multidimensional Personality Questionnaire. *Psychological Assessment*, *14*(2), 150–163. 10.1037//1040-3590.14.2.150. 12056077 10.1037//1040-3590.14.2.150

[CR41] Pincham, H. L., Bryce, D., Kokorikou, D., Fonagy, P., & Pasco Fearon, R. M. (2016). Psychosocial intervention is associated with altered emotion processing: An event-related potential study in at-risk adolescents. *Plos One*, *11*(1), 1–16. 10.1371/journal.pone.0147357. 10.1371/journal.pone.0147357 PMC4726793 26808519

[CR40] Pincham, H. L., Bryce, D., Fonagy, P., & Pasco Fearon, R. M. (2019). Psychosocial intervention in at-risk adolescents: Using event-related potentials to assess changes in decision making and feedback processing. *European Child & Family Psychiatry*, *28*, 223–236. 10.1007/s00787-018-1167-3. 10.1007/s00787-018-1167-3 29802517

[CR42] Preece, D. A., Becerra, R., Robinson, K., & Gross, J. J. (2020). The emotion regulation questionnaire: Psychometric properties in general community samples. *Journal of Personality Assessment*, *102*(3), 348–356. 10.1080/00223891.2018.1564319. 30714818 10.1080/00223891.2018.1564319

[CR43] Puetz, V. B., Viding, E., Gerin, M. I., Pingault, J., Sethi, A., Knodt, A. R., Radtke, S. R., Brigidi, B. D., Hariri, A. R., & McCrory, E. (2020). Investigating patterns of neural response associated with childhood abuse v. childhood neglect. *Psychological Medicine*, *50*(8), 1398–1407. 10.1017/S003329171900134X. 31190662 10.1017/S003329171900134X

[CR44] Riepenhausen, A., Wackerhagen, C., Reppman, Z. C., Deter, H., Kalisch, R., Veer, I., & Walter, H. (2022). Positive cognitive reappraisal in stress resilience, mental health, and well-being: A comprehensive systematic review. *Emotion Review*, *14*(4), 310–331. 10.1177/17540739221114642.

[CR45] Sach, M., Enge, S., Strobel, A., & Fleischhauer, M. (2018). MPQ Control (versus Impulsivity) and need for Cognition: Relationship to behavioral inhibition and corresponding ERPs in a Go/No-Go task. *Personality and Individual Differences*, *121*, 200–205. 10.1016/j.pai.2017.04.005.

[CR46] Selye, H. (1936). A syndrome produced by diverse nocuous agents. *Nature*, *138*, 32. 10.1176/jnp.10.2.230a 9722327

[CR47] Shackman, J. E., Shackman, A. J., & Pollak, S. D. (2007). Physical abuse amplifies attention to threat and increases anxiety in children. *Emotion*, *7*, 838–852. 10.1037/1528-3542.7.4.838. 18039053 10.1037/1528-3542.7.4.838

[CR48] Sill, J., Popov, T., Schauer, M., & Elbert, T. (2020). Rapid brain responses to affective pictures indicate dimensions of trauma-related psychopathology in adolescents. *Psychophysiology*, *57*(1), 1–12. 10.1111/psyp.13353. 10.1111/psyp.13353 PMC6991163 30807662

[CR49] Simonetti, A. (2020). Electroencephalography and Childhood Trauma in *Childhood Trauma in Mental Disorders*.

[CR50] Teicher, M. H., Andersen, S. L., Polcari, A., Anderson, C. M., Navalta, C. P., & Kim, D. M. (2003). The neurobiological consequences of early stress and childhood maltreatment. *Neuroscience & Biobehavioral Reviews*, *27*, 33–44. 10.1016/s0149-7634(03)00007-1. 12732221 10.1016/s0149-7634(03)00007-1

[CR51] Teicher, M. H., Dumont, N. L., Ito, Y., Vaituzis, C., Giedd, J. N., & Andersen, S. L. (2004). Childhood neglect is associated with reduced corpus callosum area. *Biological Psychiatry*, *56*, 80–85. 10.1016/j.biopsych.2004.03.016. 15231439 10.1016/j.biopsych.2004.03.016

[CR52] van Bodegom, M., Homberg, J. R., & Henckens, M. J. A. G. (2017). Modulation of the hypothalamic-pituitary-adrenal axis by early life stress exposure. *Frontiers in Cellular Neuroscience*, *11*(87), 1–33. 10.3389/fncel.2017.00087. 28469557 10.3389/fncel.2017.00087 PMC5395581

[CR53] van Cauwenberge, V., Van Leeuwen, K., Hoppenbrouwers, K., & Wiersema, J. R. (2017). Developmental changes in neural correlates of cognitive reappraisal: An ERP study using the late positive potential. *Neuropsychologia*, *95*, 94–100. 10.1016/j.neuropsychologia.2016.12.015. 27988161 10.1016/j.neuropsychologia.2016.12.015

[CR54] Vythilingam, M., Heim, C., Newport, J., Miller, A. H., Anderson, E., Bronen, R., Brummer, M., Staib, L., Vermetten, E., Charney, D. S., Nemeroff, C. B., & Bremner, J. D. (2002). Childhood trauma associated with smaller hippocampal volume in women with major depression. *American Journal of Psychiatry*, *159*, 2072–2080. 10.1176/appi.ajp.159.12.2072. 12450959 10.1176/appi.ajp.159.12.2072 PMC3230324

[CR55] Weinberg, A., Venables, N. C., Proudfit, G. H., & Patrick, C. J. (2015). Heritability of the neural response to emotional pictures: Evidence from ERPs in an adult twin sample. *SCAN*, *20*, 424–434. 10.1093/scan/nsu059. 10.1093/scan/nsu059 PMC4350478 24795435

[CR56] Yehuda, R., Halligan, S. L., & Grossman, R. (2001). Childhood trauma and risk for PTSD: Relationship to intergenerational effects of trauma, parental PTSD, and cortisol excretion. *Development and Psychopathology*, *13*, 733–753. 10.1017/s0954579401003170. 11523857 10.1017/s0954579401003170

[CR57] Zuckerman, M. (1991). *Psychobiology of personality*. Cambridge University Press.

